# Homeostasis and function of regulatory T cells (Tregs) *in vivo*: lessons from TCR-transgenic Tregs

**DOI:** 10.1111/imr.12165

**Published:** 2014-04-09

**Authors:** Kesley Attridge, Lucy S K Walker

**Affiliations:** 1 Kennedy Institute of Rheumatology, University of OxfordOxford, UK; 2 Institute for Immunity and Transplantation, University College London Medical SchoolLondon, UK

**Keywords:** Tregs, immune regulation, tolerance, TCR-transgenic, Treg proliferation, Treg function

## Abstract

The identification of CD25 and subsequently Forkhead box protein 3 (Foxp3) as markers for regulatory T cells (Tregs) has revolutionized our ability to explore this population experimentally. In a similar vein, our understanding of antigen-specific Treg responses *in vivo* owes much to the fortuitous generation of T-cell receptor (TCR)-transgenic Tregs. This has permitted tracking of Tregs with a defined specificity *in vivo*, facilitating analysis of how encounter with cognate antigen shapes Treg homeostasis and function. Here, we review the key lessons learned from a decade of analysis of TCR-transgenic Tregs and set this in the broader context of general progress in the field. Use of TCR-transgenic Tregs has led to an appreciation that Tregs are a highly dynamic proliferative population *in vivo*, rather than an anergic population as they were initially portrayed. It is now clear that Treg homeostasis is positively regulated by encounter with self-antigen expressed on peripheral tissues, which is likely to be relevant to the phenomenon of peripheral repertoire reshaping that has been described for Tregs and the observation that the Treg TCR specificities vary by anatomical location. Substantial evidence has also accumulated to support the role of CD28 costimulation and interleukin-2 in Treg homeostasis. The availability of TCR-transgenic Tregs has enabled analysis of Treg populations that are sufficient or deficient in particular genes, without the comparison being confounded by repertoire alterations. This approach has yielded insights into genes required for Treg function *in vivo*, with particular progress being made on the role of *ctla-4* in this context. As the prospect of manipulating Treg populations in the clinic becomes reality, a full appreciation of the rules governing their homeostasis will prove increasingly important.

This article is part of a series of reviews covering Regulatory Cells in Health and Disease appearing in Volume 259 of *Immunological Reviews*.

## Introduction

Despite the theoretical capacity to form >10^15^ unique αβ T-cell receptors (TCRs) 1, humans contain only around 10^12^ T cells 2, indicating that only a small fraction of potential TCR specificities is deployed at any one time. Protective immunity against a wealth of unpredictable infections therefore relies on a high level of TCR cross-reactivity 3,4 that is beginning to be documented experimentally 5. In light of this understanding, the simplistic notion that T cells are purged of self-reactive specificities during thymic development becomes untenable; removing all cells with the potential to cross-react with self-antigens would leave us with a dangerously narrow protective repertoire. In parallel with this realization, support has grown for the existence of additional tolerance mechanisms that might compensate for the inherent limitations of negative selection. Indeed, the concept of dominant tolerance, whereby T cells with regulatory function actively control the self-reactive T cells that enter the peripheral repertoire, is now well recognized 6–10. Considerable research effort has focused on unraveling the identity and characteristics of the regulatory T-cell population(s) and a large body of knowledge has now been gathered in this area. One approach that has proved informative is the use of regulatory T cells (Tregs) expressing transgenic TCRs, permitting analysis of the antigen-specific activation of this population *in vivo*. Here, we review some of the insights that have been made using this approach and how it has shaped our understanding of Treg biology.

## TCR-transgenic Tregs: a window on Treg thymic selection

While the focus of this review is the homeostasis and function of Tregs in the periphery, it is important to first briefly consider Treg thymic selection. Study of TCR-transgenic Tregs has been formative in shaping many of our current ideas about how Tregs are generated intrathymically. In fact, the important observation that thymocytes could be directed to differentiate into Treg in response to thymic self-antigen expression was made using a TCR-transgenic system with specificity for influenza hemagglutinin (HA) 11. This paradigm was subsequently shown to hold true for other transgenic TCR/cognate antigen double-transgenic systems 12,13. For example, DO11.10 TCR-transgenic mice on a recombinase activating gene-deficient (RAG^−/−^) background fail to develop Tregs 14; however, transgenic provision of ovalbumin (OVA) in the thymus permits Treg generation in these mice 13. In the latter experiments, OVA was expressed under the control of the rat insulin promoter (rip-mOVA), which is known to drive expression of functionally significant levels of OVA intrathymically 15, like the endogenous insulin promoter 16. Interestingly, RAG-sufficient DO11.10 TCR-transgenic mice were found to select a small number of Tregs in the absence of thymic OVA expression 13,14,17, but these Tregs were enriched for endogenous TCRα chains 13,17 consistent with the use of an alternative TCR to support their differentiation. Thus, T cells with TCRs that cannot recognize intrathymic antigen (DO11.10/RAG^−/−^) were precluded from differentiating into Treg, but provision of relevant antigen in the thymus (DO11.10^+^ rip-mOVA^+^ RAG^−/−^), or the potential to recognize intrathymic antigen using non-transgenic TCR chains (DO11.10/RAG^+^) permitted Treg generation.

Leading on from the use of TCRs specific for model antigens, a subsequent wave of experiments employed transgenic TCRs that were derived from naturally occurring Tregs 18,19. These experiments reinforced the role of TCR specificity in Treg thymic selection and suggested a model in which intraclonal competition limits the number of T cells of a given specificity that can develop into Tregs 18,19. Whether such niche constraints restrict Treg differentiation in the context of a diverse repertoire of precursors remains less clear 20.

## Tregs as a highly proliferative population

### Lack of Treg anergy *in vivo*

**Figure 1:**
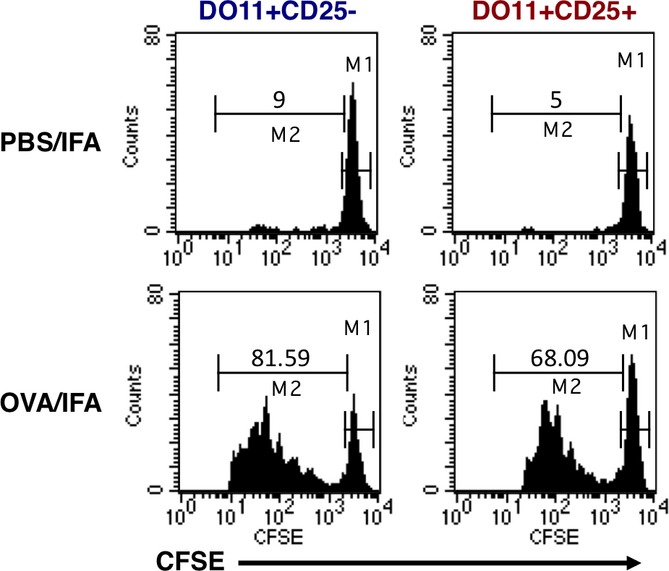
Proliferation of T-cell receptor-transgenic Tregs *in vivo* after immunization with cognate antigen. Conventional or regulatory T cells were purified from DO11 × rip-mOVA mice, CFSE-labeled, and adoptively transferred into BALB/c recipients. Recipient mice were injected subcutaneously with PBS or OVA emulsified in incomplete Freund's adjuvant. Three days later, the proliferation of DO11^+^ T cells in draining LN was assessed.

Early analysis of mature Tregs using *in vitro* assays identified a striking lack of proliferation following TCR engagement, leading to the characterization of this population as naturally anergic 14,21,22. In fact, anergy came to be known as one of the defining features of Treg populations. The generation of mice bearing TCR-transgenic Tregs provided the first opportunity to monitor Treg behavior *in vivo*, using clonotypic antibodies to track a discrete cohort of adoptively transferred Tregs responding to a specific antigen. Surprisingly, this approach revealed a remarkable capacity for Tregs to proliferate when responding to cognate antigen *in vivo*. Accordingly, OVA-specific Tregs mounted a robust proliferative response to OVA protein in incomplete Freund's adjuvant (IFA) 13 (*Fig. *1), while HA-specific TCR-transgenic Tregs proliferated and accumulated in the draining lymph node (LN) following subcutaneous immunization with HA peptide in IFA 23.

The Tregs utilized in the above experiments derived from double-transgenic mice expressing the TCR transgene in conjunction with its relevant antigen under ubiquitous (phosphoglycerate kinase-HA) or tissue-specific (rip-mOVA) control. In both sets of double-transgenic mice, CD25^+^ cells expressing the TCR transgene were detectable in the thymus and peripheral lymphoid organs 13,23 and exhibited typical Treg features including the presence of intracellular cytotoxic T-lymphocyte antigen-4 (CTLA-4) and low levels of surface interleukin 7 receptor α (IL-7Rα) expression 13. These experiments were performed prior to the development of Forkhead box protein 3 (Foxp3) staining protocols, but several lines of evidence argued against the CD25^+^ cells being activated conventional T cells rather than Tregs. Notably, the extensive proliferative response of this population was uncoupled from production of cytokines such as IL-2, interferon-γ (IFNγ), and IL-4 13,23, and instead IL-10 production was observed in one of the studies 23. In addition, while TCR-transgenic conventional T cells upregulated CD40L following antigen encounter, this response was completely lacking in the TCR-transgenic CD25^+^ fraction 13. Perhaps, the most compelling demonstration that the TCR-transgenic CD25^+^ cells were in fact Tregs was that despite their capacity to proliferate *in vivo*, they were strictly anergic when assessed *in vitro* and elicited robust suppression in standard co-culture assays 13,23. Thus, TCR-transgenic Tregs recapitulated the *in vitro* behavior previously ascribed to this subset, yet permitted new insights into the antigen-responsiveness of this population *in vivo*.

In addition to responding to protein antigen emulsified in adjuvant, TCR-transgenic Tregs were also shown to proliferate following provision of peptide-pulsed bone marrow-derived dendritic cells (DCs) 24. In the latter study, subcutaneous injection of OVA-pulsed DCs triggered the proliferation of OVA-specific Tregs in the draining LN, leading to an eight- to 10-fold expansion over the course of 3 days. Elegant studies in which mice were engineered to transgenically express TCR chains from a scurfy mouse-derived CD4^+^ T cell also revealed robust proliferation within the Foxp3^+^ compartment 25, demonstrating that the phenomenon extended beyond the extensively utilized TCRs specific for model antigens.

**Figure 2:**
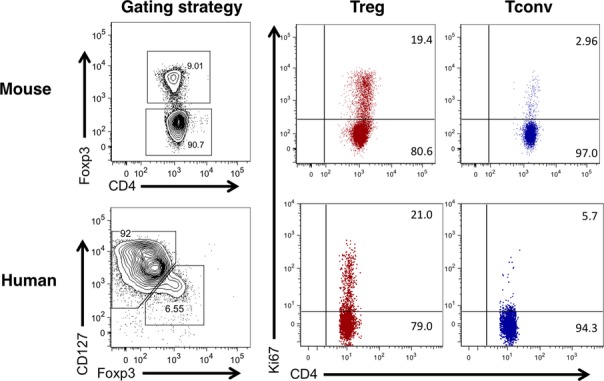
Analysis of mouse and human Treg proliferation by Ki67 staining. Tregs from the LN of a BALB/c mouse or from human blood were stained with Ki67. Murine Tregs were defined as CD4^+^ Foxp3^+^. Human Tregs were defined as CD4^+^ CD127^low^ Foxp3^+^.

Validation that TCR-transgenic Tregs were accurately mimicking the proliferative capacity of normal polyclonal Tregs came from experiments in which CD4^+^ CD25^hi^ CD62L^hi^ cells were carboxyfluorescein succinimidyl ester (CFSE)-labeled and adoptively transferred into congenically distinct recipient mice 26. Thirty-five days later, approximately 70% of the donor Tregs had divided, most of them more than six times. Interestingly, the CD25^hi^ phenotype of the injected Treg population was remarkably stable, with approximately 90% of the cells retaining high CD25 expression even 70 days following transfer. This is consistent with the subsequent demonstration that Treg Foxp3 expression is also very stable under physiologic conditions 27. Bromodeoxyuridine (BrdU) labeling experiments further supported the notion that Tregs represent a highly proliferative population in normal mice, with Tregs consistently incorporating higher levels of this thymidine analog than conventional T cells 26,28. In line with this, the proportion of CD4^+^ Foxp3^+^ Tregs staining positive for the proliferation marker Ki67 is consistently higher than that of the conventional T-cell fraction (*Fig. *2), and is still higher if Tregs are rendered deficient in the lipid phosphatase PTEN 29. Further insight into the biochemical control of Treg proliferation was recently gleaned by analysis of animals in which raptor, an obligatory component of the mammalian target of rapamycin complex 1 (mTORC1) complex, was selectively deleted in Tregs 30. Raptor-deficient Tregs showed a striking lack of proliferation, compared with control Tregs, when cell trace labeled and adoptively transferred into normal recipients. These experiments established a critical role for raptor/mTORC1 signaling in Treg proliferation, with the mevalonate pathway proving particularly important 30.

Experiments examining the proliferative capacity of human Tregs have largely recapitulated findings in mice (*Fig. *2). Despite *in vitro* anergy 31,32, when analyzed *ex vivo*, on average 22% of blood CD4^+^ CD25^+^ CD127^lo^ Foxp3^+^ T cells stained positive for the proliferation marker Ki67 compared with 5% of the CD4^+^ CD25^int^ cells and 2% of the CD4^+^ CD25^−^ cells 33. This high proliferative capacity was maintained over a period of 3–4 months in longitudinally tracked individuals 33. Detailed characterization of human Treg populations by the Sakaguchi group has clarified that the proliferative fraction resides within the CD45RA^−^ Foxp3^hi^ ‘activated Treg’ population 34. Interestingly, recent analysis indicates that Tregs isolated from human secondary lymphoid organs show evidence of an even greater proliferative capacity than those found in blood, with approximately 60% staining positive for Ki67 compared with around 20% in blood-derived Tregs 35. Collectively, these studies have radically changed our view of Tregs from an anergic and unresponsive population to a highly dynamic one, equipped to respond rapidly to homeostatic and antigenic cues 36.

### Treg proliferation in infection, autoimmunity, and cancer

In addition to proliferating in the steady state, it is now well established that a variety of stimuli can enhance Treg proliferation. These include a wide range of immunological perturbations including exposure to infectious agents. For example, it has been demonstrated that Tregs proliferate and accumulate at sites of *Leishmania major* infection, with up to 80% of Tregs from such sites showing the capacity to respond specifically to *Leishmania*-infected DCs *ex vivo* 37. Similarly, infection of mice with *Mycobacterium tuberculosis* (Mtb) was shown to trigger proliferation of pathogen-specific TCR-transgenic Tregs 38. Subsequent studies by the same group used tetramers to show that endogenous *Mtb*-specific Tregs also proliferated and expanded in the pulmonary lymph node of infected mice. Importantly, Treg accumulation was short-lived due to selective loss of these cells via an IL-12 and Tbet-dependent mechanism, thereby lifting regulation during the latter stages of infection 39. Viruses can also trigger the expansion of Tregs 40–42, and in one case, this was shown to occur by a superantigen-dependent pathway 43. Similarly, Tregs are known to proliferate in response to helminths with the proportion of Foxp3^+^ Tregs incorporating BrdU in the pleural cavity increasing from approximately 20% to over 50% following *Litomosoides sigmondontis* infection 44. In a separate study, Tregs expanded in response to *Heligmosomoides polygyrus* infection in an inducible costimulatory (ICOS)-dependent manner, and this was shown to reflect a role for ICOS in supporting the survival of divided Tregs, rather than an obligate role in proliferation itself 45.

In the context of autoimmune disease, Tregs have been shown to exhibit heightened proliferation in the peripheral blood of individuals with systemic autoimmunity 46 and at the site of inflammation in tissue-specific autoimmunity. For example, Tregs from the synovial fluid of arthritis patients showed substantially higher proliferation than those in peripheral blood 47, and increased Treg proliferation has been documented in the inflamed CNS of mice with EAE 48 and the pancreas of BDC2.5 NOD mice 49. In the latter model, mice express a transgenic TCR comprising rearranged TCRα and β genes from a diabetogenic T-cell clone isolated from a non-obese diabetic (NOD) mouse 50. Both Treg and effector T cells infiltrate the islets in BDC2.5 NOD mice, and disease incidence is low unless Treg development is precluded by introduction of the Foxp3 *scurfy* mutation 51. Tregs infiltrating the pancreas of BDC2.5 NOD mice were shown to incorporate higher levels of BrdU than conventional T cells present at this site 49. Consistent with this observation, Tregs infiltrating the islets in non-TCR-transgenic NOD mice showed increased staining for Ki67 compared with conventional T cells; intriguingly, this was the case in new onset disease but not in prediabetic animals 52. The loss of pancreas-resident Tregs following administration of the chemotherapeutic alkylating agent cyclophosphamide 53 may in part reflect its ability to efficiently inhibit Treg proliferation 54.

Tregs have long been recognized to be overrepresented in tumors 55, prompting interest in their proliferation at such sites. In the context of B16F10 and 4T1 tumors, analysis of tumor-draining LNs showed that Tregs proliferated substantially more than effector CD4^+^ or CD8^+^ T cells 56. Tregs infiltrating brain tumors in a mouse model of glioblastoma showed markedly increased proliferation compared with their Foxp3-negative counterparts 57. Furthermore, analysis of carcinogen-induced sarcomas in mice revealed that over 60% of the tumor-infiltrating Tregs incorporated BrdU following a 3-day pulse, clearly illustrating the high proliferative potential of the tumor-resident Treg population 58. Expansion of intratumoral Tregs is thought to reflect the marked proliferation of a few dominant clones 59 and could be driven in part by the high levels of TGFβ that typifies the tumor microenvironment 60 or by factors associated with angiogenesis such as vascular endothelial growth factor 61.

The discovery that Tregs can proliferate in the steady state has been rapidly followed by an appreciation that this proliferative response can be significantly augmented in a variety of medically relevant settings including infection, autoimmunity, and cancer. Such expansion could potentially serve to bolster the Treg population during immunological insults, such that they are poised to efficiently terminate immune responses 62.

## Factors controlling peripheral Treg homeostasis

### Role of TCR signaling in shaping Treg homeostasis

**Figure 3:**
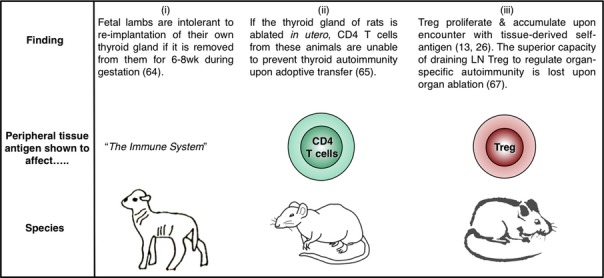
Role of peripheral self-antigen in Treg homeostasis – 2 decades of progress. (i) Early experiments (1989) in fetal lambs showed that the continued presence of the thyroid gland was necessary for maintenance of immunological tolerance toward this organ. The cell type mediating such tolerance was not defined. (ii) Studies in the rat (1999) showed that ablation of the thyroid gland compromised the ability of peripheral CD4^+^ T cells to regulate autoimmune thyroiditis. (iii) Experiments in mice (2003, 2004, 2009) showed that Tregs proliferate in response to tissue-expressed self-antigen; Tregs isolated from draining LNs had a heightened capacity to regulate autoimmune organ destruction in a manner that was lost upon organ ablation.

A major force that shapes the homeostasis of Treg populations in the periphery is signaling through the TCR. The seeds of this idea were sown by Peter McCullagh 63, who hypothesized that within any given T-cell clone, both ‘high’ and ‘low’ pathogenicity cells are selected in the thymus, with the latter able to regulate the pathogenicity of the former. Importantly, the low pathogenicity (regulatory) clones were postulated to have a short duration of survival following release from the thymus unless exposed to their specific ligand 63. The experimental basis for McCullagh's hypothesis was the observation that temporary removal of the thyroid gland in gestating lambs triggered its destruction upon re-implantation, but rejection did not occur if part of the gland was allowed to remain in the animal 64. Further support for the notion that Treg homeostasis was controlled by access to peripheral self-antigen (*Fig. *3) emerged from studies in rats; ablation of thyroids *in utero* rendered peripheral CD4^+^ T cells incapable of preventing thyroid autoimmunity, while preserving their capacity to control other autoimmune diseases 65. The implication of these data is that CD4^+^ T cells with regulatory activity fail to expand to a sufficient frequency or fail to survive, if denied the opportunity to encounter relevant autoantigen in the periphery.

The advent of TCR-transgenic Tregs permitted the direct assessment of the role of peripheral self-antigen in controlling Treg homeostasis. CFSE-labeled OVA-specific 13 or HA-specific 26 Tregs were adoptively transferred into mice expressing the relevant antigen as a tissue-specific self-protein, and the proliferative response in the draining LN was assessed a few days later. In both cases, antigen expression was directed to the pancreatic islets through use of the rat insulin promoter, and the antigen-specific Tregs were demonstrated to proliferate specifically in the pancreatic LNs but not in non-draining LNs 13,26 (*Fig. *3). Importantly, the absolute number of OVA-specific Tregs in the draining LN was higher after adoptive transfer into mice expressing OVA as a self-antigen compared with antigen-negative littermates 13, consistent with a role for peripheral antigen in positively regulating Treg homeostasis. Similarly, Tregs endowed with the capacity to regulate autoimmune ovarian disease were found to be concentrated in the ovary-draining LN 66. In fact, it was shown for a range of organ-specific autoimmune diseases that Treg activity was enriched in the autoantigen-draining LN in a manner that could be abolished by selective autoantigen ablation 67. These data add a CD4^+^ CD25^+^ Foxp3^+^ dimension to the thyroid ablation studies performed a decade earlier 65 that examined the regulatory capacity of the CD4^+^ population as a whole (*Fig. *3). As LNs draining the site of autoantigen expression have been shown to be key sites for the priming of autoreactive pathogenic T cells 68,69, it appears that regional LNs are actively involved in both the generation and suppression of tissue-specific autoimmune responses.

The considerable proliferative capacity of Tregs *in vivo* begs the question of which cell type supports such a response. In this regard, most attention to date has focused on DCs. DCs have been shown to be capable of driving Treg proliferation both *in vitro* and *in vivo* 24, particularly those bearing a CD8α-negative phenotype 70. DCs are also required for the expansion of Tregs in the setting of chronic LCMV infection 43. Ablation of DCs in mice expressing diphtheria toxin receptor under the control of the CD11c promoter significantly reduced Treg proliferation 71, although it should be noted that mice constitutively lacking DCs from birth showed only a marginal reduction in Treg numbers 72. Nevertheless, augmenting the DC population by exogenous Flt3 ligand administration significantly increases the Treg population, even in thymectomized mice, arguing for effects on peripheral homeostasis rather than thymic generation 73. Further support for an influential role for DCs in Treg homeostasis derives from the observation that Treg numbers correlate with the number of major histocompatibility complex (MHC) class II-expressing DCs 74. The requirement for DCs to express MHC class II to influence Treg homeostasis 74 is consistent with a role for Treg TCR signaling and fits with the observation that Tregs rendered p56(Lck)-deficient are impaired in their proliferative response *in vivo* 75.

The above observations suggest a model in which the size of the peripheral Treg niche is set by the availability of MHC class II-expressing DCs bearing relevant peptides. This model is supported by the demonstration that wildtype Tregs, presumably selected on a diverse array of peptides in the thymus, failed to divide in the periphery if transferred to mice in the which the MHC class II molecules were loaded with a single peptide, CLIP 76. In contrast, Tregs selected within a thymic environment expressing only CLIP proliferated to a greater extent in the periphery of these animals, consistent with having more Tregs-bearing TCRs that recognize this peptide 76. Artificially supplementing TCR engagement by administration of anti-CD3 permits enlargement of the Treg population by driving additional Tregs into cycle, offering further support for the involvement of TCR signaling in setting the size of the peripheral Treg niche 77.

The proliferation 13,26 and selective accumulation 66,67,78 of Tregs in LN draining sites of self-antigen expression implies that Treg with a given TCR specificity is not uniformly distributed throughout the body but rather are subject to local homeostatic control. In line with this idea, by sequencing TCRα chains in mice with a fixed TCRβ chain, the Hsieh laboratory 79 showed that the TCR repertoire of Tregs varies substantially according to anatomical location. This variation differs from similar analysis of the naive T-cell population, which revealed little repertoire skewing between anatomical sites 79, consistent with the free recirculation of naive T cells between secondary lymphoid organs.

Lymphoid organs aside, there is an increasing appreciation that Tregs can be found embedded within tissues themselves (e.g. within adipose tissue) 80 and that the repertoire of these Tregs may be distinctive 81. Now that the existence of tissue-resident memory T cells has been clearly documented in humans 82, understanding whether discrete populations of Treg police each of these niches and defining the rules governing the maintenance of such populations will be an important aim.

### Role of IL-2 in peripheral Treg homeostasis

It is well accepted that the cytokine most influential in Treg homeostasis is IL-2. The immunoregulatory role of this cytokine first came to light with the observation that mice deficient in IL-2 signaling exhibit polyclonal expansion of lymphocytes with tissue infiltration, autoantibody production, anemia, and in some cases, inflammatory bowel disease 83–85. Available evidence, albeit limited, suggests that IL-2Rα deficiency also causes a similar phenotype in humans, with lymphadenopathy, anemia, and multi-organ lymphocytic infiltration 86. The prospect that this might reflect impairment of regulatory cells was raised by mixing experiments in which the presence of IL-2-sufficient T cells curbed the uncontrolled expansion of those rendered IL-2 deficient 87. The advent of CD25 as a marker of the Treg population permitted a more refined version of this experiment in which CD4^+^ CD25^+^ cells were shown to prevent lethal autoimmunity following adoptive transfer into IL-2Rβ-deficient mice 88. Along similar lines, CD4^+^ CD25^+^ cells prevented disease caused by the introduction of IL-2Rα^−/−^ bone marrow into rag-deficient hosts 89. Thus, the dysregulated immunity in mice lacking IL-2 signaling could be attributed to a failure to maintain CD4^+^ CD25^+^ Tregs.

The central role for IL-2 in Treg homeostasis and metabolic fitness has since been clearly demonstrated 90,91. IL-2 has been shown to reinforce qualitative aspects of the Treg program in addition to simply conferring a survival advantage, as Tregs that lack IL-2 signaling express low levels of numerous markers associated with the Treg phenotype (CTLA-4, CD39, CD73) 92. Interestingly, the role of the microRNA miR155 in promoting Treg homeostasis is thought to reflect its ability to inhibit SOCS1, thereby sensitizing Treg to IL-2 signaling 93. Along similar lines, Tregs are characterized by low SOCS3 expression, compared with conventional T cells, further increasing their IL-2 responsiveness 94.

Elegant experiments using bone marrow chimeric mice revealed that the size of the peripheral CD4^+^ CD25^+^ Treg compartment is directly related to the number of conventional T cells capable of producing IL-2 95. Consistent with this scenario, neutralizing IL-2 with antibodies reduces the steady state *in vivo* proliferation of Treg in normal mice 71,96. Conversely, acute Treg ablation triggers a dramatic increase in the proliferation of those Tregs remaining (from approximately 20% to 70% Ki67^+^), and this increase correlates with increased plasma levels of IL-2 97. The pivotal role of IL-2 in setting the size of the peripheral Treg compartment has led to therapeutic IL-2 administration being tested in mouse models of autoimmunity 52,98,99 and subsequently in several human clinical trials 100–103.

The coupling of Treg numbers to conventional T-cell IL-2 production 95 laid the foundation for the notion that Treg homeostasis and function can be ‘boosted’ by the conventional T-cell compartment 104. Accordingly, experiments with TCR-transgenic T cells and Tregs sharing the same specificity revealed that T-cell activation triggers a feedback loop whereby suppressive capacity is augmented hand in hand with the conventional T-cell response 104. One manifestation of this is the striking observation that when conventional T cells are activated by antigen *in vivo*, the early wave of IL-2 produced serves primarily to activate Treg in the locality 105. However, IL-2 cannot explain the whole of the boost effect, and a role for TNFα in augmenting Treg proliferation was also identified 104. Intriguingly, recent data suggest that in certain settings, Treg function may also be boosted by engagement of their surface Nrp1 by the ligand Sema4a 106. The feedback loops operating to ensure appropriate suppression during immune responses are only now beginning to emerge and alterations in these may well underlie the immune imbalance observed in the context of autoimmunity.

### Role of CD28 in Treg homeostasis

One of the earliest pathways identified in our quest to understand Treg homeostasis was costimulation through CD28. The surprising observation that autoimmune diabetes was exacerbated in mice lacking CD28, or its ligands CD80 and CD86, was reconciled by the discovery that Tregs were dramatically reduced in these animals 107. Tregs were defined on the basis of CD25 expression in the latter study, but the deficit of Tregs in CD28 knockout mice was later confirmed using Foxp3 staining 108. The proliferation of CFSE-labeled polyclonal Treg adoptively transferred into syngeneic mice could be blocked by antibodies to CD80 and CD86 109, and this was associated with a decrease in Treg number and also a loss in the intensity of CD25 expression 109. The latter was subsequently attributed to CD28 deficiency in the conventional T-cell compartment, presumably resulting in decreased IL-2 availability 110. The contribution of NF-κB-inducing kinase (NIK) to CD28 signaling 111 may provide a potential explanation of why NIK-deficient Tregs exhibit a defect in peripheral homeostasis 112.

Further evidence for the importance of costimulatory signals in Treg homeostasis arose from analysis of CTLA-4-deficient mice that have a lymphoproliferative phenotype 113,114 driven by excessive CD28 signaling 115. These animals exhibit increased Treg proliferation and an augmented Treg population 116,117 consistent with the role of CD28 in promoting Treg proliferation. Likewise, short-term blockade of CTLA-4 in wildtype mice increases Treg proliferation 116,118,119, an effect that is recapitulated by administration of anti-CTLA-4 antibody to humans 120. Given the importance of CD28 in driving Treg proliferation and the role of CTLA-4 in limiting CD28 engagement 121–123, the Treg proliferation induced in CTLA-4^−/−^ mice and by anti-CTLA-4 antibody likely results from enhanced CD28 signaling due to increased availability of costimulatory ligands.

The recent generation of mice in which CD28 can be inducibly deleted has allowed further exploration of the role of this receptor in Treg homeostasis 110. Accordingly, tamoxifen-induced CD28 deletion was shown to trigger a dramatic reduction in Treg frequencies as early as 1 week after treatment 110. The decrease in Tregs was also observed in thymectomized mice and was associated with reduced Ki67 staining, implying a crucial role for CD28 in peripheral Treg maintenance. To home in on the role of CD28 specifically in Tregs, the Turka group 124 generated an animal model in which CD28 was selectively deleted from Foxp3-expressing cells. Mice with a floxed CD28 gene were crossed with Foxp3-YFP-Cre mice, such that Foxp3 promoter activity was linked both to yellow fluorescence protein (YFP) expression and excision of the floxed CD28 gene. A decrease in the proportion of Foxp3^+^ Treg was observed within the CD4 single-positive population in the thymus 124, in line with the known role of CD28 in promoting thymic selection of Tregs 108,109,125. However, the peripheral Treg compartment was replete in these animals, consistent with the notion that peripheral Treg homeostasis can be uncoupled from thymic output 89. Intriguingly, the animals lacking CD28 in Tregs developed autoimmune symptoms over time, primarily focused on the liver and skin, supporting a requirement for CD28 in Treg function 124. In female mice heterozygous for Foxp3-Cre in which approximately half the Tregs delete CD28 (due to random inactivation of the X-chromosome), CD28-dependent effects on Treg homeostasis were revealed. Accordingly, the CD28-sufficient Tregs in these animals incorporated significantly more BrdU than those lacking CD28 in the same animal 124. The competitive advantage conferred by Treg CD28 expression was further revealed by mixed bone marrow transfers into irradiated recipients: when analyzed 6 months later, the entire Treg compartment appeared to derive from CD28-sufficient rather than CD28-deficient cells, while the rest of the hematopoietic compartment remained of mixed origin 124.

The early studies showing that DCs could drive Treg proliferation *in vivo* highlighted the importance of CD86/CD80 expression for this function; OT-II TCR-transgenic Treg proliferated robustly in the presence of OVA peptide-pulsed lipopolysaccharide-matured DCs, but the response was reduced by around 70% if the DCs derived from CD80/CD86 knockout mice 24. The key roles for DCs 71,73,74 and the CD80/CD86 axis 107,109 in maintaining Treg homeostasis begged the question of whether DCs represent the critical source of costimulatory ligands for Treg *in vivo*. This issue was elegantly explored by experiments in which DC-deficient (CD11c:DTA) bone marrow and CD80/CD86-deficient bone marrow were mixed and used to reconstitute lethally irradiated wildtype mice 126. The only DCs able to develop in the resulting chimeras lacked expression of CD80 and CD86, but other cell types (such as B cells) expressed these ligands at normal levels. In the absence of DC-expressed CD80/CD86, Tregs developed normally in the thymus, but their numbers were markedly reduced in the periphery 126. This suggests that DCs are a crucial source of CD28 ligands for maintaining peripheral Treg homeostasis.

The role of CD28 in positively regulating Treg homeostasis also appears to hold true in humans 127. Accordingly, the proliferative response of human Tregs cultured for 6 days with allogeneic mature DCs is largely inhibited by blocking anti-CD86 antibody 128. In addition, IDO activation was shown to modulate proliferation of human Tregs in the context of mixed lymphocyte reaction responses in a manner dependent on CD80/CD86 129. Thus, CD28 costimulation represents a primary pathway for promotion of Treg homeostasis in both mouse and human.

### Apoptosis of peripheral Tregs

Given the considerable proliferation of Tregs evident *in vivo*, one might expect this population to be continually increasing in size. As this is not the case, it follows that Treg proliferation must be countered by apoptosis. Early work by Taams *et al*. 32 identified that blood CD4^+^ CD25^+^ cells had lower levels of Bcl2 than their CD25^−^ counterparts and were more prone to undergo apoptosis upon cytokine withdrawal. The propensity of murine Tregs to undergo cytokine-withdrawal apoptosis and the importance of common γ-chain cytokines in countering this process was subsequently demonstrated 130. Intriguingly, it has recently been shown that Foxp3 itself can exhibit pro-apoptotic activity, increasing the activity of Puma and Bim and repressing Bcl2 expression in thymic Treg precursors, such that these cells must compete for cytokine signals to ensure their survival 131. IL-2 is normally critical for generating a replete peripheral Treg compartment, but this requirement can be circumvented if Treg apoptosis is prevented by Bim deficiency 132.

The importance of apoptosis in setting the size of the peripheral Treg niche has recently been demonstrated by the generation of mice that selectively lack the pro-apoptotic molecules Bak1 and Bax in Tregs; these mice show increased peripheral accumulation of Tregs, despite normal thymic development 97. A surprise finding from this study was that the anti-apoptotic protein responsible for promoting survival in the Treg compartment is Mcl-1 97 rather than Bcl-2 or Bcl-XL, as has been tacitly assumed for years. Thus, the decreased level of Bcl2 observed in apoptosis-prone Tregs may actually be correlative rather than causal.

Experiments in mice suggest that the propensity of Tregs to apoptose may decline with age in a manner that is associated with decreased Bim expression. The latter conclusion was based on the observation that Bim levels were markedly lower in Tregs from 20-month-old mice compared with 1.5-month-old animals 133; how this relates to Treg homeostasis in aging humans remains to be established.

## Use of TCR-transgenic Tregs to probe suppressive mechanisms *in vivo*

It is increasingly clear that Tregs can call upon a vast array of mechanisms to maintain dominant tolerance 134,135. These are suggested to encompass use of granzyme B 136, CD73/CD39 137, CTLA-4 138,139, TGFβ 140,141, IL-10 142, and IL-35 143. Tregs have also been demonstrated to act as an ‘IL-2 sink’ 144–146 and to induce the expression of enzymes that consume essential amino acids 147, in both cases depriving conventional T cells of essential growth factors. Moreover, Tregs are thought to interact with multiple target populations to effect suppression, including conventional T cells and antigen-presenting cells.

The role of CTLA-4 in Treg-mediated suppression has been a matter of contention for more than a decade 108,148. The issue first came to light after the demonstration that unlike conventional CD4^+^ T cells, Tregs constitutively express CTLA-4 138,139,149. Many approaches have since been adopted in an effort to clearly define the function of Treg-expressed CTLA-4, an endeavor that has been complicated by a lack of clarity on the basic nature of CTLA-4-mediated inhibition. In addition, the lethal lymphoproliferative syndrome observed in CTLA-4^−/−^ mice 113,114 has rendered data obtained from these animals difficult to interpret. However, recent studies using antigen-specific Tregs have proved insightful. Indeed, our own laboratory has overcome some of the issues outlined above by using CTLA-4^−/−^ mice expressing the DO11.10 transgenic TCR on a RAG^−/−^ background. As the only TCR expressed in these mice is directed against a non-self-antigen, T cells retain a naive phenotype and the severe lymphoproliferative disease characteristic of CTLA-4^−/−^ mice is notably absent 150. Because Treg development is known to require cognate antigen expression in the thymus, crossing these mice with those expressing OVA intrathymically due to the rip-mOVA transgene permits the induction of Tregs with a single specificity that can be studied over the long term 116. An added benefit of this system is that the CD28 pathway is unaffected, unlike in systems where CTLA-4 immunoglobulin fusion protein (CTLA-4-Ig) or blocking antibodies against CD80 or CD86 is used to prevent lymphoproliferative disease.

We utilized Tregs from these mice in an adoptive transfer model of type-1 diabetes and were able to show that while CTLA-4-sufficient Tregs were effective at suppressing disease onset, lack of CTLA-4 expression specifically within the Tregs compartment prevented suppressive function, with all recipients rapidly becoming hyperglycemic 116. This was the first time that Treg populations with an identical specificity and affinity for antigen (conferred by their DO11.10/RAG^−/−^ status), either expressing or lacking CTLA-4, had been directly compared. The importance of CTLA-4 expression for Treg suppression of diabetes was consistent with the immune dysregulation triggered by Treg-specific deletion of the *ctla-4* gene 117 and has since been confirmed in another diabetes system where disease is driven by T cells responding to an endogenous pancreatic self-antigen 151.

Precisely how CTLA-4 contributes to Treg suppressive capacity is still not fully resolved. The first model put forward to account for CTLA-4-dependent cell-extrinsic function – that is the ability of CTLA-4 expressed on one cell to control the response of another cell – involved CTLA-4 ‘reverse signaling’ through CD80/CD86 on APCs. CTLA-4-Ig 152 or CTLA-4-expressing Tregs 153 were shown to activate the suppressive enzyme IDO (indoleamine 2,3-dioxygenase) in APCs, with the ensuing local tryptophan depletion leading to inhibition of T-cell proliferation. However, others were unable to replicate the induction of IDO activity in APCs by CTLA-4-Ig 154 and abatacept, a CTLA-4-Ig fusion protein used clinically, was shown to suppress T-cell responses without upregulating IDO 155. Moreover, when the response of DCs to abatacept was assessed by Affymetrix microarray, minimal gene changes were observed and none at all if belatacept, a CTLA-4-Ig molecule with higher affinity for ligands, was used 156. Strikingly, a commercially available CTLA-4-Ig protein that retains full Fc-dependent effector functions induced robust gene changes in DCs 156. Among the genes upregulated was IFNγ, which had been shown to mediate CTLA-4-Ig-dependent induction of IDO 152. These findings raise the possibility that some of the data generated with CTLA-4-Ig fusion proteins might reflect Fc-dependent signaling rather than reverse signaling through CD80/CD86.

An alternative model of cell-extrinsic CTLA-4 function has emerged involving the downregulation of CD80/CD86 on APCs. In common with the reverse signaling model, this involves the APCs being rendered immunosuppressive, but this time as a result of impaired costimulatory ligand expression. The downregulation of CD80/CD86 expression on APCs by incubation with CTLA-4-expressing cells has been noted in numerous studies 116,117,157–163 and in many cases is blocked by anti-CTLA-4 antibody 116,159,162,163 or CTLA-4 deficiency 117. In collaboration with the Sansom group 164, we identified a novel molecular mechanism by which CTLA-4 can downregulate CD80/CD86 that involves removal of these ligands from APCs by a process of trans-endocytosis. Using TCR-transgenic Tregs, we demonstrated that this process could occur *in vivo*, establishing that adoptively transferred Tregs could acquire GFP-tagged CD86 molecules from host APCs 164. Using CTLA-4^−/−^ TCR-transgenic Tregs that, we had shown, lack the capacity to regulate diabetes 116, we demonstrated that ligand trans-endocytosis *in vivo* was strictly dependent on CTLA-4 expression 164. Interestingly, we found that activated CTLA-4-expressing conventional T cells were also capable of trans-endocytosis, consistent with the recent demonstration that conventional T cells can utilize CTLA-4 in a cell-extrinsic manner *in vivo* 165,166.

In addition to exploring the role of CTLA-4, TCR-transgenic Tregs have also been used to study the involvement of the TGFβ pathway in Treg suppression. Although the importance of TGFβ for maintaining immune homeostasis is unquestioned, whether Tregs need TGFβ for their suppressive function and whether conventional T cells need to perceive TGFβ to be suppressed has been unclear. Using the BDC2.5 TCR, Ishigame *et al*. 157 investigated the necessity of TGFβR-mediated signals for Treg function. In this study, BDC2.5 T cells were adoptively transferred into SCID/NOD mice, with diabetes effectively prevented by co-transfer of Foxp3-RFP^+^ BDC2.5 Tregs. Repeating these co-transfers using the same Treg lacking TGFβRII revealed that TGFβ signaling via this receptor is not required for Treg function, as all recipients remained disease-free 167. Likewise Tregs lacking TGFβRII expression retained the capacity to regulate colitis, resulting in colon histopathology indistinguishable from that of mice treated with TGFβRII-sufficient Tregs 168. This differs from results seen when effector T cells were rendered insensitive to TGFβ using a dominant negative form of the TGFβRII; in mouse models of diabetes 169 and colitis 170, effector cells that were unable to respond to TGFβ failed to be appropriately controlled by Tregs. Curiously, in the colitis model, when TCR-transgenic Tregs (DO11.10) that were either sufficient or deficient for TGFβ1 were compared, no difference was observed in their ability to suppress pathology 170 and yet suppression was abrogated by anti-TGFβ antibody in both cases. This observation suggests that successful suppression of a destructive immune response by Tregs can require TGFβ even when the Tregs themselves are not producing this cytokine. Using T cells lacking TGFβRII expression, rather than those expressing dominant negative TGFβRII, it was recently shown that conventional T cells do not require the capacity to respond to TGFβ signaling for suppression to occur *in vivo* 168, mirroring results obtained earlier using *in vitro* assays 171. While at face value, these data are hard to reconcile, one possible interpretation is that TGFβ is not a direct mediator of Treg function *per se* but is involved in generating an environment permissive of suppression. Thus, TGFβ availability would support Treg function, without being an absolute requirement for it. Since strong TCR signaling can release T cells from Treg suppression 172,173, the observation that TGFβ decreases T-cell sensitivity to TCR stimulation 168 could potentially be important in this regard.

## Insights into peripheral Treg induction

In addition to their valuable contribution to our understanding of thymus-derived Tregs (tTregs), TCR-transgenic systems have helped to shed light on the biology of T cells induced to express Foxp3 in the periphery (pTreg) [for Treg nomenclature, see Abbas *et al*. 174]. The potential to start with a mono-specific conventional T-cell population free from Tregs is a major advantage of such systems. In elegant experiments that utilized the known specificity of the 5C.C7 TCR, the Allison laboratory 175 demonstrated that optimal Foxp3 induction in adoptively transferred CD4^+^ T cells occurred in response to low doses of high-affinity ligand. Furthermore, the small population of cells that did express Foxp3 in response to low affinity peptide was found to have contracted by day 5 post immunization and did not persist over the long term 175. These data mirror findings in the thymus suggesting a requirement for relatively strong TCR stimulation for efficient Treg generation 11.

To determine whether Tregs arose in the periphery in response to a pancreatic self-antigen, Wong *et al*. 176 utilized transgenic mice expressing the diabetogenic BDC2.5 TCR. Using TCR-based lineage tracing, the authors showed that conversion from the conventional to the regulatory T-cell compartment occurred only at very low frequencies both within the pancreas and its draining lymph node 176. In addressing the same question, the Ziegler laboratory 177 adoptively transferred naive CD4^+^ Foxp3^−^ GFP^−^ T cells from DO11.10 TCR-transgenic RAG^−/−^ bone marrow chimeric mice into RAG^−/−^ recipients expressing OVA under the control of the rat insulin promoter. In this system, any Treg occurring in recipient mice must have arisen from Foxp3^−^ donor cells. At 18 days post cell transfer, Tregs were observed in both the spleen and pancreatic lymph node, affording long-term protection against diabetes onset 177. The contrasting results observed between these reports are likely due to differences in the experimental systems utilized, including the presence of existing Treg populations in the BDC2.5 TCR study and the use of lymphopenic recipients in the DO11.10 TCR study. In this regard, it has recently been shown that thymus-derived and peripherally induced Tregs share a common peripheral niche, and that the former can outcompete the latter when both are present together 178.

TCR-transgenic systems have also been used to probe the differential characteristics of thymic-derived tTregs versus peripherally induced pTregs. By adoptively transferring OT-II TCR-transgenic CD4^+^ Foxp3^−^ cells into congenic hosts, the Shevach laboratory 179 demonstrated that unlike their thymic-derived counterparts, pTregs did not express the transcription factor Helios when induced with low-dose OVA. However, the validity of Helios as a marker of the tTreg population is now disputed 180–182. It has recently been proposed that tTregs and pTregs can be effectively distinguished based on their expression of the receptor Neuropilin-1 (Nrp-1). To examine this possibility, two groups used TCR-transgenic mice on a RAG-deficient background, which generate progeny lacking tTregs. Yadav *et al*. 183 found that despite the lack of Tregs in the thymus, 1B3 TCR-transgenic RAG^−/−^ mice demonstrated the emergence of a peripheral population of CD4^+^ Foxp3^+^ cells within roughly 3 weeks of birth. Transcriptional analyses comparing these Tregs with those from 1B3 TCR-transgenic animals, which generate tTregs normally, showed lower expression of Nrp-1 by pTregs 183. Along these same lines, Weiss *et al*. 184 used DO11.10 TCR-transgenic RAG^−/−^ mice, which similarly lack tTregs, to show that pTregs generated in the gut mucosa in response to OVA antigen express lower levels of Nrp-1 mRNA than the total Tregs pool in wildtype BALB/c mice.

TCR-transgenic systems have also facilitated functional comparison of thymus-derived Tregs and peripherally induced Tregs. In one study, IL-10 differentiated DCs (DC10) were used to generate induced Tregs either *in vitro* or *in vivo* and these were directly compared with tTregs in a model of airway inflammation 185. The presence of the OT-II TCR transgene controlled for antigen specificity between the different Treg populations. Following adoptive transfer of pTregs or tTregs into recipients at day 46 post sensitization with OVA, pTregs were found to be superior at ameliorating airway hyper-reactivity and controlling IgE levels 185. More work is required to delineate the extent to which thymic-derived and peripherally induced Treg populations play distinct versus overlapping roles in immune regulation.

## Bypassing Treg suppression *in vivo*

Given the importance of Tregs in controlling the activity of self-reactive T cells, there has been much interest in whether defects in Treg number or function might underlie the development of autoimmune diseases. Using a TCR-transgenic system in which TS1 TCR^+^ cells induce arthritis in response to PR8 HA expressed as a self-antigen, the Caton laboratory 186 identified a key role for the Treg TCR repertoire in determining the efficacy of suppression. As such, TS1 TCR^+^ Tregs suppressed the clonotypic Tconv that shared their specificity but failed to inhibit arthritis due to their inability to suppress bystander T cells of different TCR specificities 186. Along similar lines, Treg populations with a lower TCR diversity were shown to be poorer at controlling pathology in a model of graft-versus-host disease 187. Treg repertoire constraint may well underlie the development of autoimmune diseases in mice subjected to thymectomy 3 days following birth 188; pTregs can be detected in these animals 189 and have the potential to elicit regulatory function 190, yet they fail to prevent the emergence of autoimmune pathology. It is now known that Treg repertoires undergo ‘reshaping’ in the periphery 191,192, likely reflecting encounter with self or environmental antigens. While Treg cohorts with a lower TCR diversity are also subject to peripheral ‘reshaping’, their lack of breadth ultimately results in a heightened risk of autoimmunity 193. Thus, conventional T-cell responses can escape appropriate control in cases where the TCR repertoire of the Treg population is not sufficiently diverse.

**Figure 4:**
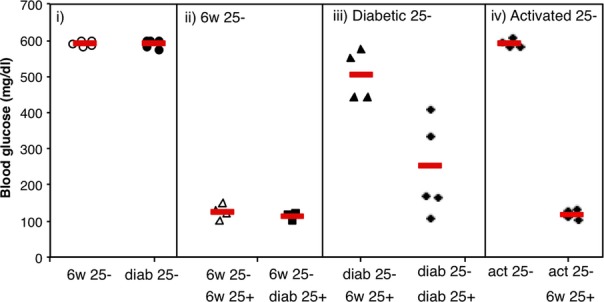
Tregs from diabetic mice are functional *in vivo,* but conventional T cells show resistance to suppression. Conventional OVA-specific T cells from healthy 6-week-old or diabetic 12- to 13-week-old (diab) DO11 × rip-mOVA mice were adoptively transferred into rip-mOVA/RAG^−/−^ recipients. Where indicated, OVA-specific Tregs from healthy or diabetic animals were co-injected. (i–iii) Tregs from diabetic mice were highly effective at controlling diabetes; however, conventional T cells from diabetic mice showed resistance to suppression. (iv) OVA-specific T cells that had been activated for 4 days *in vitro* with 1 μg/ml OVA peptide retained sensitivity to suppression, suggesting that resistance was not simply a consequence of activation. Figure adapted from Clough *et al*. 78.

In the setting of diabetes, despite no obvious deficit of Treg numbers (reviewed in 194), we were keen to explore whether Tregs might be functionally impaired in diabetic animals. Using the DO11 x rip-OVA model of type-1 diabetes, we found that Tregs in diabetic animals appear to be fully functional, even showing somewhat enhanced suppressive capacity 78 (*Fig. *4). We observed that conventional CD4^+^ T cells (Tconv) taken from either prediabetic or diseased animals induced diabetes with equivalent efficacy when transferred into Rip-mOVA RAG^−/−^ recipients but showed differing sensitivity to regulation. Co-transfer of Tregs with Tconv cells from prediabetic mice was highly successful at inhibiting diabetes but was ineffective at suppressing disease induced by Tconv cells from diabetic animals 78. These data suggested that during progression to overt diabetes, conventional T cells in this model develop resistance to Treg-mediated suppression. Similar results from the Mathis laboratory have shown that Tregs from NOD mice show no defect in suppressive capacity compared to those from wildtype animals; rather, NOD Tconv are less prone to suppression than wildtype Tconv 195 consistent with earlier observations in this model 196,197. Importantly, analysis of lymphocyte populations from Type 1 diabetes patients has confirmed that Tconv cells can exhibit resistance to Treg suppression in this setting 198,199.

In the DO11 × rip-mOVA mouse model, levels of IL-21 mRNA increased in Tconv isolated from the draining pancreatic lymph node as this population acquired resistance to Treg suppression 78. IL-21 was shown to interfere with Treg suppression *in vitro*, and this was associated with inhibition of Tconv IL-2 production, thereby starving Tregs of a critical survival factor 200. Interestingly, IL-21 could substitute for the lack of IL-2 in the Tconv population, permitting their responses to proceed unimpaired 200. Other reported examples of cytokines capable of counteracting Treg suppression include IL-2 201, IL-6 202, IL-7 and IL-15 203, IL-1 and IL-18 204, and TNFα 205. Interestingly, although these cytokines utilize a variety of pathways to counteract suppression, IL-27 has also been shown to impair Treg homeostasis in a similar fashion to IL-21, by inhibiting IL-2 production *in vivo* 206. In other cases, cytokine signaling may simply substitute for CD28 costimulation, thus bypassing the need for Tconv to compete with constitutively CTLA-4-equipped Tregs for costimulatory ligands.

## Concluding remarks

The tools of modern immunology, most notably TCR transgenesis and fate-mapping reporters (e.g. Foxp3-GFP), have revolutionized our capacity to study Treg biology. The key feature of both approaches is that they facilitate study of Tregs in a physiologic setting *in vivo*. In particular, the serendipitous generation of animal models in which Tregs express transgenic TCRs has permitted analysis of cohorts of antigen-specific Tregs responding to immunized-antigen or tissue-expressed self-antigen in a whole animal. The lessons that we have learned from such analyses serve to underline the fundamental similarity between conventional and regulatory T cells, with both populations feeding off TCR stimulation, CD28 costimulation, and cytokine signaling. A key difference appears to be the constitutively active state of the Treg population, epitomized by higher levels of proliferation that presumably reflect encounter with self-antigen. Given the remarkable proliferative capacity, site-specific accumulation, and dynamic repertoire reshaping that characterizes the Treg population in the periphery, it is somewhat incongruous that they were initially defined as anergic. A better understanding of the parameters that determine the size of the peripheral Treg niche and the division of labor between thymus-derived and peripherally derived populations should provide guidance on the best strategies to manipulate these cells clinically.
